# A Fast and Real‐Time Machine Vision Evaluation System for Size Grading of *Agaricus bisporus* Based on Video

**DOI:** 10.1002/fsn3.71328

**Published:** 2026-01-08

**Authors:** Qiyang Shui, Fajun Miao, Senping Liu, Liang Cao, Huanyu Jiang, Jinzhu Lu

**Affiliations:** ^1^ Modern Agricultural Equipment Research Institute, School of Mechanical Engineering Xihua University Chengdu China; ^2^ School of Mechanical Engineering Xihua University Chengdu China; ^3^ Chengdu Academy of Agriculture and Forestry Sciences Chengdu China; ^4^ College of Biosystems Engineering and Food Science Zhejiang University Hangzhou China; ^5^ School of Aeronautics and Astronautics Xihua University Chengdu China

**Keywords:** *Agaricus bisporus*, factory‐scale grading, postharvest grading, YOLOv5

## Abstract

Automatic grading in factory production is a key component for improving production efficiency and ensuring product quality. As one of the most widely consumed edible mushrooms in the world, the cap diameter of *Agaricus bisporus* is a key factor affecting the market selling price. In addition to the high labor cost, manual grading is subjective and prone to human assessment errors. Therefore, this study proposes a video‐based fast real‐time machine vision evaluation system for size grading of *Agaricus bisporus* mushrooms. The designed grading system consists of a conveyor belt to achieve transportation of *Agaricus bisporus* and an overhead color camera to perform size quality assessment. Considering deployment on edge devices, we have optimized the YOLOv5 model for lightweight performance by adopting ShuffleNetV2 as the backbone network, significantly improving the model's lightweight performance and processing speed. At the same time, we investigated the balance between transport speed and recognition accuracy to ensure that the system maintains a high recognition rate while producing efficiently. The results show that the size of the improved model is reduced by 86.1%, FPS is increased by 9.8%, and mAP is 97.1%. The experimental field results show that the grading can still be performed at a high speed with a transmission speed of 130.17 mm/s. The grading speed can reach 1066.67 mushrooms/min with an accuracy of 97.87%, and it also has strong adaptability and stability in low resolution and low light conditions. The video‐based model can detect *Agaricus bisporus* mushrooms that are always in motion, which is more suitable for practical detection than a single image‐based detection model.

## Graphical abstract

In this study, to address the problem of high cost and strong subjectivity of artificial grading of *Agaricus bisporus*, we propose a fast real‐time video‐based machine vision grading system, which achieves high‐speed grading of 1066.67 mushrooms per minute with an accuracy of 97.87% by lightweighting the YOLOv5 model and optimizing the balance between the transmission speed and the recognition precision, and exhibits strong adaptability in low‐resolution and low‐light conditions and stability.

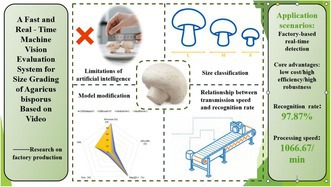

## 1 Introduction

As one of the most widely consumed edible mushrooms globally, *Agaricus bisporus* has gained increasing popularity among consumers due to its nutritional benefits, including low‐fat content, high protein, polysaccharides, multivitamins, and multiminerals (Yan et al. 2023). According to the Food and Agriculture Organization of the United Nations (FAO), the global production of *Agaricus bisporus* has increased significantly, rising from 3.8 million tons to 5 million tons over the past decade. This growth in production is accompanied by heightened demands for quality control throughout the mushroom production process. The cap size of fresh *Agaricus bisporus* is a critical factor in determining its tenderness and maturity, which directly impacts its culinary quality. Overly mature mushrooms tend to have a less desirable texture when cooked. Additionally, *Agaricus bisporus* exhibits a high metabolic rate and respiratory intensity, coupled with the absence of a protective surface structure, making it highly susceptible to microbial infection. This susceptibility often leads to browning, decay, and spoilage, significantly reducing the mushroom's shelf life (Zhou et al. 2022). Furthermore, the market price of *Agaricus bisporus* is closely tied to the cap size of fresh mushrooms. To maximize economic benefits, there is a growing trend in the industry to implement rapid grading and packaging of *Agaricus bisporus* immediately after harvesting, ensuring that the mushrooms meet market standards before distribution. This approach not only enhances product quality but also aligns with the increasing consumer demand for fresh and high‐quality mushrooms. At present, *Agaricus bisporus* mushrooms need to go through the process of soil removal, root trimming, grading, pre‐cooling, etc. (NY/T 1790–2009) after harvesting, of which the grading link is labor‐intensive, costly, and dependent on manual labor, which is inefficient and of different standards (Zhong et al. 2024). With the growth of production and quality requirements, manual grading has become a bottleneck. The development of machine vision technology for agricultural product grading brings new ideas. Numerous studies have been applied to the grading of agricultural products such as apples (Sofu et al. 2016), pears (Song et al. 2024), mangoes (Raghavendra et al. 2020), figs (Baigvand et al. 2015), jujubes (Pham et al. 2025), potatoes (Su et al. 2018), carrots (Deng et al. 2021), vegetables (Liu et al. 2025), rice (Sun et al. 2024), etc., and the accuracy and efficiency have been significantly improved.

For the research application of edible mushrooms, scholars have used image analysis algorithms to extract features from mushrooms to identify features such as size, color, shape and freshness (Tian et al. 2024). For example, Wang et al. (2023) proposed a mushroom grade recognition method based on a modified VGG network (D‐VGG), which utilizes the Fishhawk Optimization Algorithm (OOA) to improve the computational efficiency of Otsu threshold binarization in order to obtain a complete sample of the mushroom contour, and replaces the fully‐connected layer with a globally averaged pooled layer to achieve the recognition of six mushroom grades, with a grading accuracy of 96.21%. Lu et al. (2019) developed a deep learning convolutional neural network based system to localize mushrooms, which performs well on mushroom image measurement. Ketwongsa et al. (2022) used convolutional neural network (CNN) and regional convolutional neural network (R‐CNN) to classify five common poisonous edible mushrooms in Thailand and compare the test time and accuracy of different training models to distinguish edible and poisonous edible mushrooms. Meanwhile, Yang et al. (2025) detected and identified the browning of *Agaricus bisporus* with the help of a portable hyperspectral camera and machine learning strategy. Wei et al. (2024) proposed a postharvest low‐temperature (4°C) storage quality grading method for apricot mushrooms based on hyperspectral imaging, clustering algorithm and comprehensive evaluation, which classified the samples into high, medium and poor categories based on 11 physicochemical indexes of K‐means algorithm and principal component analysis, and the final identification rate was 91.58%. In the field of computer vision in recent years, the development of target detection technology has greatly promoted the process of agricultural automation and intelligence. Among them, the YOLO (You Only Look Once) series of algorithms have attracted much attention for their efficient real‐time and accuracy (Yin et al. 2022). Various edible mushroom detection systems based on YOLO detection models have been studied by a large number of scholars. Tao et al. (2024) proposed a ReYOLO‐MSM evaluation method based on the relationship between monocular vision and mushroom rod growth to solve the problem of selective harvesting identification of mushroom rods, with a detection accuracy of 99.3% and a mAP of 98.9%. Liu et al. (2022) detected the surface texture of shiitake mushrooms using the optimized YOLOX model. Wei et al. (2022) proposed the Recursive‐YOLOv5 algorithm based on YOLOv5 to detect edible mushrooms in vertical bar scenarios with a 98% recognition rate for small targets with large resolution. Cong et al. (2023) constructed MYOLO, a lightweight model based on YOLOv3, to achieve cracked, flat, and misshapen mushroom grading. Shi, Mo, et al. (2024) and Shi, Wei, et al. (2024) improved the YOLOv8n to obtain the OMC‐YOLO model, which realized the special, first and second class grading of flat mushrooms, and the mAP50 value reached 94.95%.

At present, the sorting technology based on machine vision is mature, for its application in edible fungi sorting requires more research. Some of the existing research is analyzed as shown in Table 1, where “√” and “—” represent the types of containing and not containing. It can be seen that most of the current equipment in the field of *Agaricus bisporus* grading research regarding the use of machine vision is for single mushroom detection and grading; multiple quantities are detected only, recognized, and not graded. *Agaricus bisporus* detection based on the YOLO algorithm is mainly confined to the algorithm verification stage using static images in laboratories. It does not take into account dynamic factors in actual production (such as conveyor belt vibrations, target overlaps, and changes in lighting), and its algorithm parameters (input resolution, detection frame rate, etc.) are difficult to meet the stringent requirements of industrial production in terms of real‐time performance and stability. More critically, these systems generally overlook industrial deployment requirements: the excessive computational load of the models makes edge device deployment challenging, severely limiting the practical application value of the technology. Rapid identification and grading of large quantities are core requirements for achieving efficient, large‐scale operations in factory production.

**TABLE 1:** Existing studies.

Researchers	Multiple	Grading	Method	Accuracy
Wang et al. (2018)	—	√	Watershed algorithm	97.42%
Jiang et al. (2022)	—	√	Watershed algorithm	96.45%
Chen, Wang, et al. (2023)	√	—	Improved YOLOv5	98%
Chen, Yi, et al. (2023)	√	—	Improved watershed algorithm	95.7%
Shi, Mo, et al. 2024	√	—	Improved RTMDet	98.64%
Lu and Liaw (2020)	√	—	YOLOv3	82.7%
Yang et al. (2022)	√	—	Segmentation recognition algorithm	96%

However, vision technology grading is often accompanied by automated transport, and in addition to grading accuracy, grading efficiency should also be considered. Nowadays, automation has been applied in factory‐based production, where production lines usually need to grade and package a large number of products efficiently, and conveyors need to be set up in such a way as to ensure that production efficiency is improved without compromising quality assessment. In the study of conveyor and production efficiency, Jensen et al. (2011) proposed an empirical soft sensor model to regulate the conveyor speed with a PID controller to maintain the moisture content of paired leaf outlet in the range of 0.024–0.034 (dry basis). Yang et al. (2014) developed an on‐line visual inspection system for mining conveyor belts based on a binary belt image detection algorithm to identify longitudinal tears and belt deviations. Böhner et al. (2013) used computational fluid flow simulation to improve the problem of uneven drying in the direction of conveyor belt dryer bandwidth for system optimization. Liu et al. (2021) used a high‐speed camera and RBF neural network to remove defective bamboo parts from conveyor belts. Gao et al. (2019) proposed a non‐contact speed measurement system to measure the speed of a conveyor belt by image texture. Ma et al. (2024) designed a bionic non‐smooth structure screw conveyor to improve the performance of agricultural particles conveying. Zhao et al. (2024) improved the slide structure of a potato grading device and optimized its performance through orthogonal tests. Elwakeel et al. (2023) calibrated RGB color channels to optimize the performance of RGB‐based automatic fruit sorting sensors.

To the best of our knowledge, less research has been conducted on production grading applications for the factory transfer process, and real‐time, online quality assessment and grading of *Agaricus bisporus* mushrooms using YOLOs has not yet been performed. There is an urgent need for a mushroom system that can detect and evaluate multiple quantities and grades of mushrooms in modern *Agaricus bisporus* production to meet the factory production requirements and enhance the commercial production capacity. To this end, a real‐time, size‐automatic grading and assessment system for *Agaricus bisporus* mushrooms based on YOLOv5 is proposed. The specific objectives of this study are (1) to develop a YOLOv5‐based computer vision algorithm pipeline for real‐time assessment of *Agaricus bisporus* size and to evaluate the automatic grading performance of the proposed system; (2) to improve the detection model by lightweighting; (3) to meet the fastest transmission speed condition of the factoryization and to improve the efficiency of the factoryization production.

## 2 Materials and Methods

### 2.1 Image Data Set

#### 2.1.1 Data Set Acquisition

In order to identify and grade the quality of *Agaricus bisporus* based on machine vision, it is necessary to obtain a sufficient number of *Agaricus bisporus* detection samples.

**FIGURE 1:**
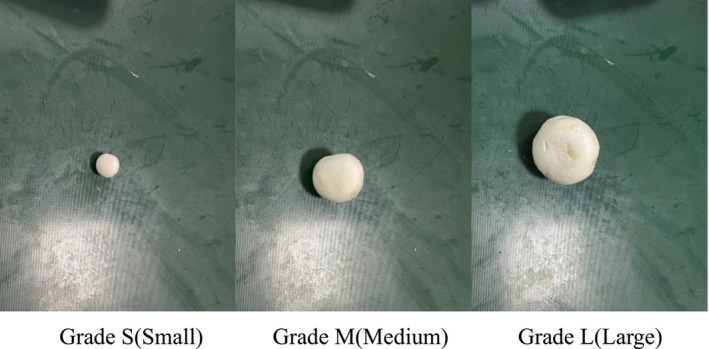
Examples of the three grades of *Agaricus bisporus*.

Through market research, it was found that the diameters of the pileus of the vast majority of *Agaricus bisporus* in the market are above 25 mm. To balance the categories in the dataset, *Agaricus bisporus* were purchased locally on multiple occasions. Initially, 423 *Agaricus bisporus* with different pileus diameters were bought from the local Walmart supermarket. Subsequently, 103 *Agaricus bisporus* were procured from the local edible–fungus cultivation base, and all these specimens were used in this experiment. Prior to the imaging experiments, each sample was precisely measured. In accordance with (NY/T 1790–2009 n.d.), a vernier caliper with a resolution of 0.01 mm was employed to measure the maximum diameter of *Agaricus bisporus* at an angle parallel to the horizontal plane. After measurement, based on the grades and specifications of *Agaricus bisporus*, the samples were classified into three grades, as shown in detail in Table 2. The three grades are Small, Medium, and Large, labeled with the codes S, M, and L respectively. Figure 1 presents examples of *Agaricus bisporus* samples of the three graded specifications.

**TABLE 2:** NY/T 1790–2009 employed in this study for grading *Agaricus bisporus*.

Grades	Diameter (D)	Label
“Small”	D < 25MM	S
“Medium”	25MM ≤ D ≤ 45MM	M
“Large”	45MM < D	L

**FIGURE 2:**
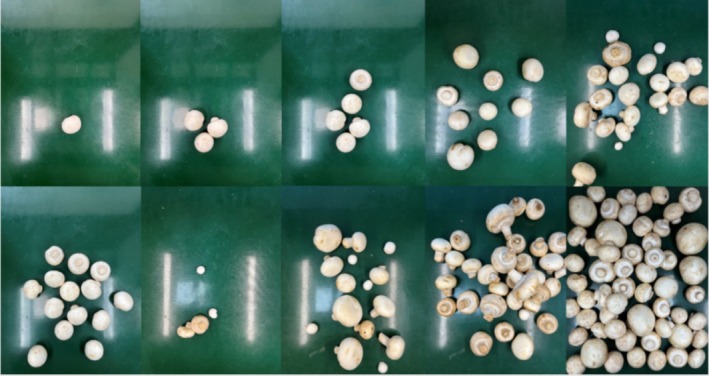
Filming of selected datasets.

This paper is a study of automatic grading during the factory production of *Agaricus bisporus*, so the data set was collected with a conveyor belt as the background and an iphone13 smartphone camera was used to capture images of each sample. Among other parameters such as focus, image capture angle, illumination conditions, and the distance between the camera and the sample were fixed. It is worth noting that in this paper, for the purpose of hierarchical identification of *Agaricus bisporus* mushrooms with multiple quantities and grades, the distance between the camera and the sample is 250 mm, the camera capture area is 0.13 m^2^, and each image can capture up to 60 *Agaricus bisporus* mushrooms. With the number of mushrooms included in each dataset ranging from 1 to 60 randomly photographed, while each dataset randomly contains mushrooms of one, two, or three grades, the final dataset of 2096 was obtained. Figure 2 shows some of the datasets photographed.

**FIGURE 3:**
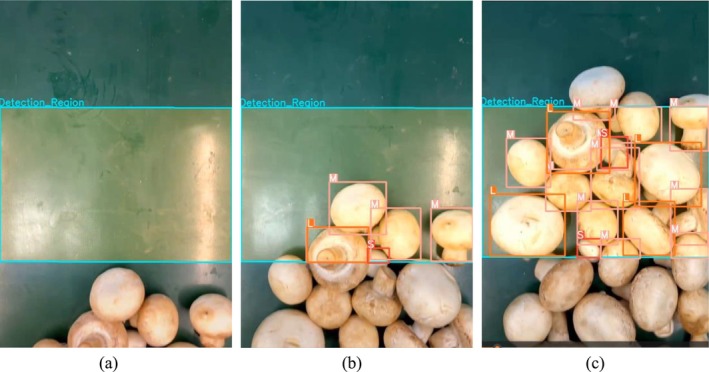
Examples of video frames for grading prediction.

In the imaging experiment, *Agaricus bisporus* samples were loaded onto the conveyor belt in a continuous, multiple quantity, and batch‐by‐batch manner at different conveyor speeds. Under the lighting conditions, the camera captured videos of the moving samples at a frame rate of 30 frames per second with a resolution of 640 × 480 pixels, and the resulting videos were saved in the MP4 (MPEG—4 Part 14) format. Figure 3 shows an example frame of one of the test videos, where (a)–(c) represent the recognition and grading process of some *Agaricus bisporus* on the conveyor belt. In total, 20 real‐time videos were captured. Among them, 12 videos were shot with random conveyor speeds, random stacking patterns, and random numbers of samples. The other 8 videos were shot with the same stacking patterns and the same number of samples, but different speeds, ensuring that the speed was the only variable. These videos form the dataset for the detailed analysis in the subsequent sections.

#### 2.1.2 Data Set Division

This study addresses factory grading and all datasets were taken with a conveyor belt as the background. Each image was manually labeled using the Labelimg data annotation tool, which included the location of the mushrooms (bounding box) and their size classes, which were labeled into three categories: S, M, and L. This division was based on the determination of the diameter and morphological characteristics of the mushrooms, which could effectively support the function of automatic grading assessment. The labeled data were saved as text files in VOC data format. In order to make the dataset more diversified, the acquisition process considered different stacking forms, occlusion, overlapping, and incomplete images, so as to ensure that the model can learn the diversity of mushroom features during the training process. To ensure the training effect and generalization ability of the model, we divided the collected image dataset according to the ratio of training set: validation set: test set: 7:2:1.

### 2.2 Detection Device

**FIGURE 4:**
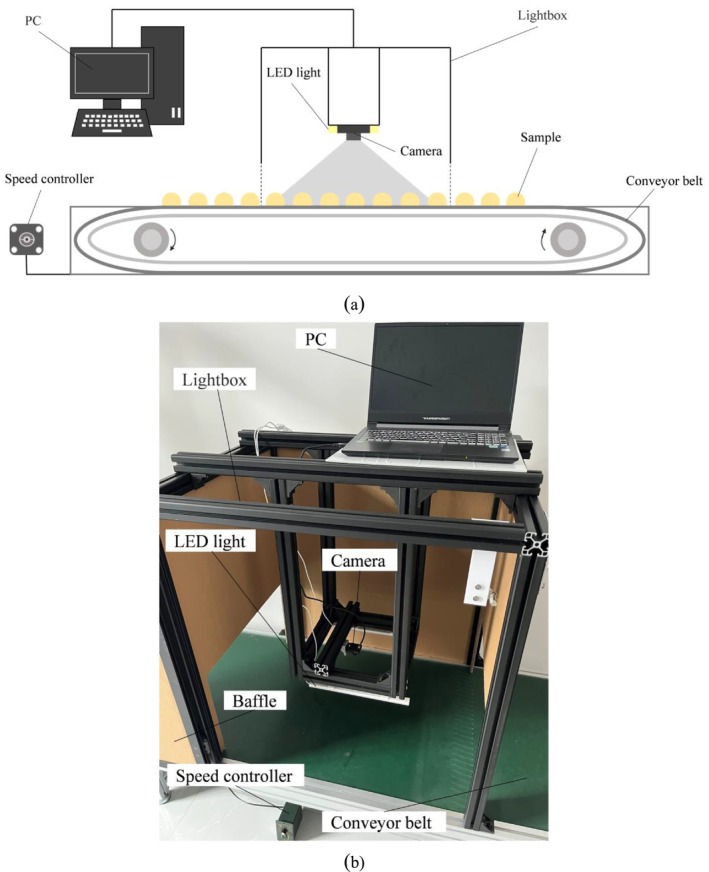
Detection device: (a) schematic diagram and (b) physical diagram.

The detection device is shown in Figure 4, where a conveyor belt is used to transport the *Agaricus bisporus*, which is moved forward by a motor. The stepping motor (42BYGH47‐401A0.55N.M) is used as a speed controller to dynamically adjust the speed of the conveyor belt according to the system requirements, thus ensuring that the *Agaricus bisporus* passes through the field of view of the camera at an appropriate speed for image acquisition. The dark box is used to avoid the interference of external light sources and ensure the stable lighting conditions in the system. The light source in the dark box is an LED light source with adjustable illumination conditions, and in order to display as many Agaricus sp. as possible in the image, the camera (V1080P‐3.0mm95°) image plane is parallel to the horizontal plane of motion of the Agaricus sp. A PC is used to acquire images from the camera in real time and process them. The image acquisition for this study and the subsequent validation were performed on this system.

### 2.3 *Agaricus bisporus* Grading Model Based on YOLOv5

The main objective of this study is to develop a computer vision algorithm based pipeline, this model needs to meet the conditions of pipeline visual inspection, such as small target, large number, fast speed, etc. for the real‐time evaluation of *Agaricus bisporus* size, for this purpose, we constructed a *Agaricus bisporus* grading model, which ensures the grading accuracy and at the same time ensures the grading efficiency. Meanwhile, this study is for grading of factoryized *Agaricus bisporus* mushroom, which needs to be deployed on mobile devices or embedded environment, so lightweight improvement of the model is necessary.

#### 2.3.1 Fast and Accurate Detection Based on YOLOv5

With the continuous development of deep learning in the field of target detection, the YOLO series always pursues the excellent trade‐off between speed and accuracy for real‐time applications. YOLOv5, as the classic version of the YOLO series, significantly improves the performance and speed of target detection by optimizing the network structure and training strategy and has become a practical application of many preferred solutions. Among them, the YOLOv5 network algorithm is an improved algorithm based on YOLOv3, which proposes a multi‐scale prediction method to detect image feature targets of different sizes at the same time, providing the best target detection domain in terms of algorithmic accuracy and runtime.

**FIGURE 5:**
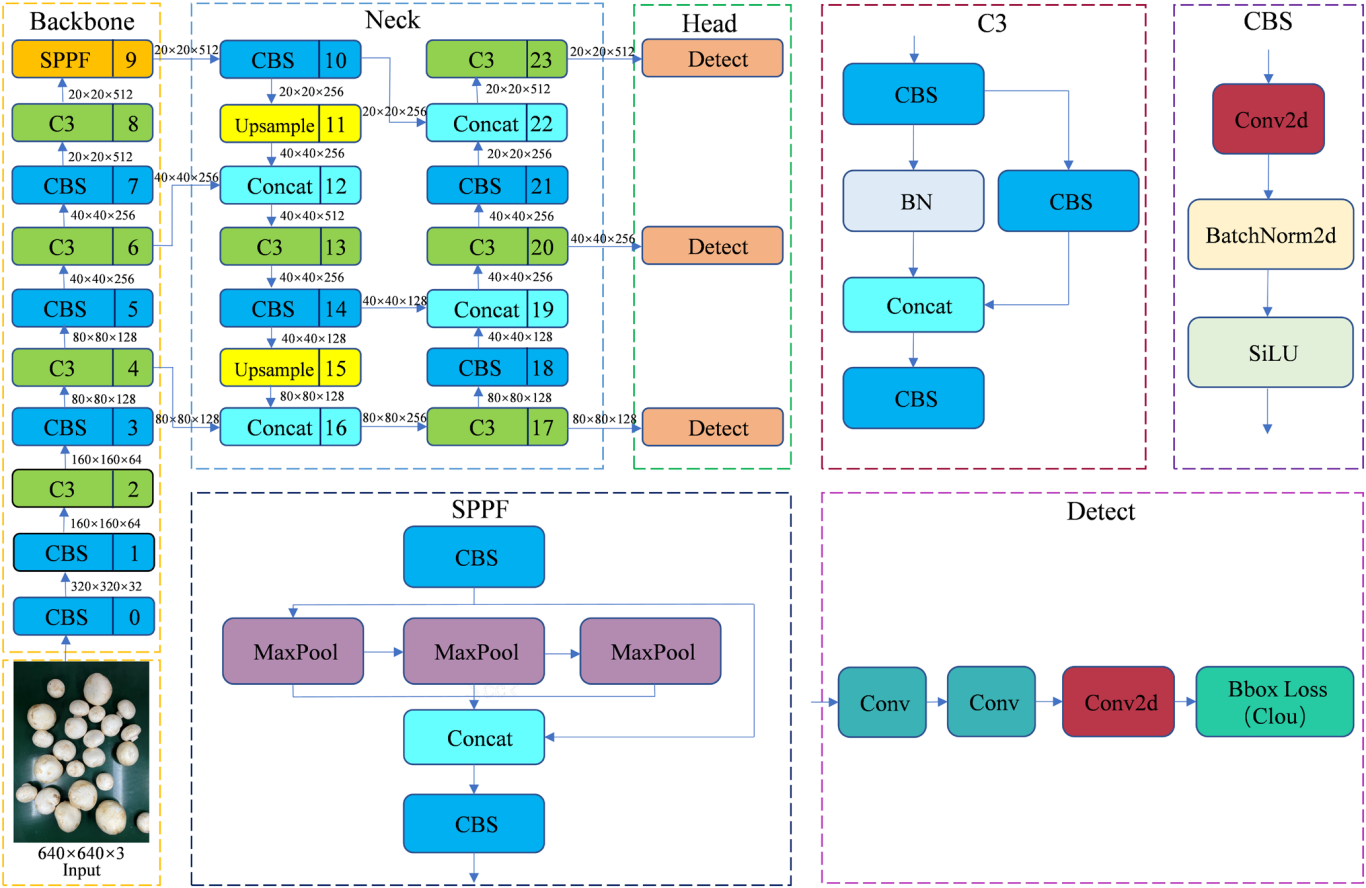
Framework of the YOLOv5s network model.

GitHub provides four network models for the YOLOv5 network, namely YOLOv5s, YOLOv5m, YOLOv5l, and YOLOv5x, with gradually increasing depth and width (Qi et al. 2022). Four versions of YOLOv5 were trained separately, with corresponding detection results shown in Table 3. The mAP values of the four models are relatively close. The mAP of the YOLOv5s model is identical to that of YOLOv5m, both at 98%, representing a 0.3% increase over the YOLOv5l and YOLOv5x models. In terms of model parameters, YOLOv5s is smaller than YOLOv5m, YOLOv5l, and YOLOv5x by 13.8, 39.1, and 79.2 MB, respectively. Additionally, the model size and GFLOPs requirements are 28.5, 79.1, and 159.4 MB smaller than YOLOv5m, YOLOv5l, and YOLOv5x, respectively, while the computational demands are 32.1G, 91.9G, and 188G lower. Therefore, considering the detection accuracy and lightweight requirements of the network, the YOLOv5s network model was adopted in this experiment. The original YOLOv5s network model is shown in Figure 5.

**TABLE 3:** Comparison of model prediction results.

Models	mAP_﹫0.5_ (%)	Parameters (M)	Size (MB)	GFlops (G)
YOLOv5s	98	7.0	14.4	15.8
YOLOv5m	98	20.8	42.2	47.9
YOLOv5l	97.7	46.1	92.8	107.7
YOLOv5x	97.7	86.2	173.1	203.8

#### 2.3.2 Improved Lightweight YOLOv5 Model

**FIGURE 6:**
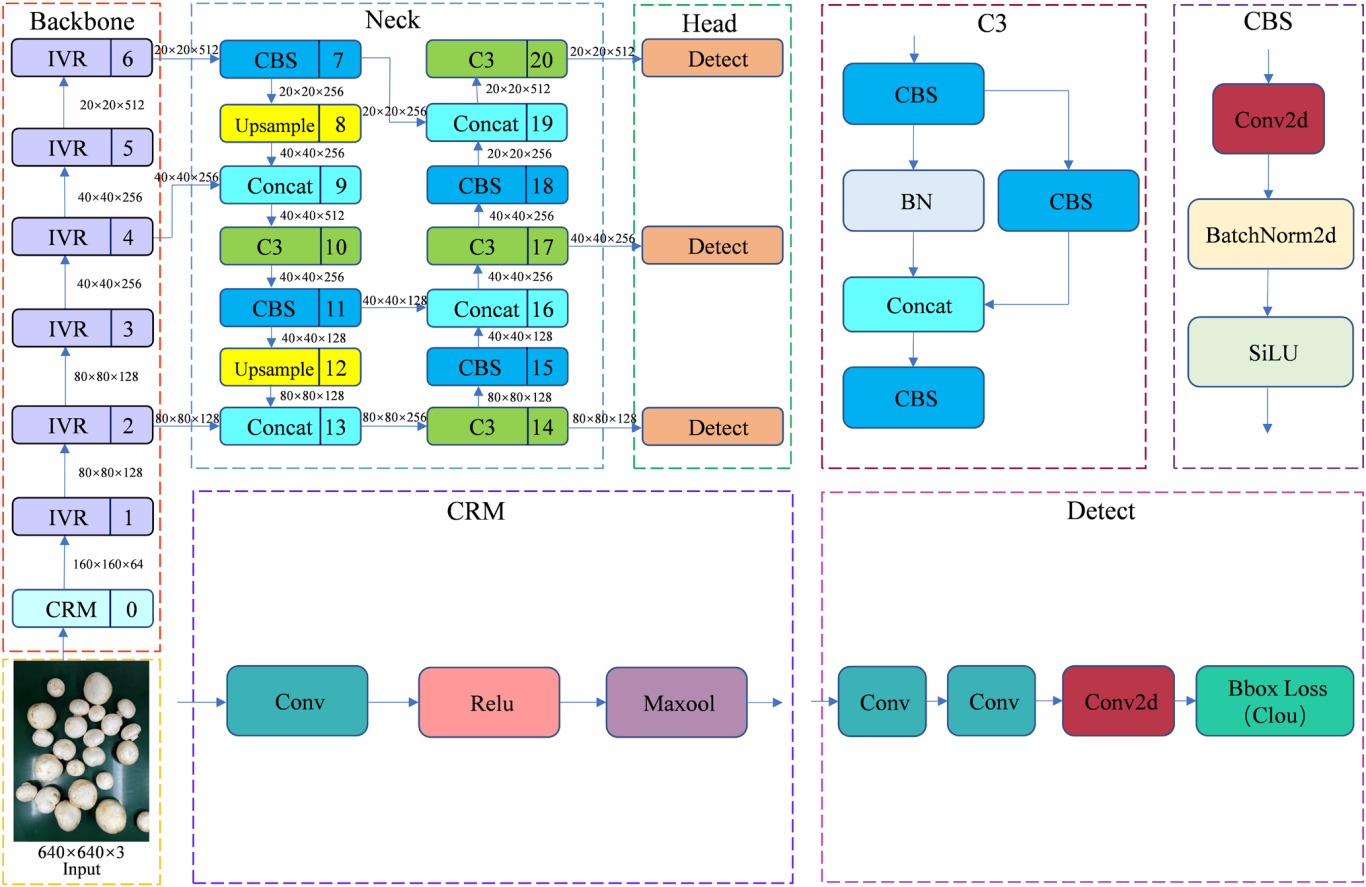
Framework of the improved YOLOv5s network model.

CSPDarknet53 (a variant of CSPNet) and Spatial Pyramid Pooling‐Fast (SPPF) module form the Backbone part of YOLOv5. CSPDarknet53 is mainly used for feature extraction, which extracts the spatial information of an image layer‐by‐layer by means of multiple convolutional layers, batch normalization layers, and activation functions. SPPF is an improvement of Spatial Pyramid Pooling (SPP), which aims to improve the efficiency of feature extraction and has good results in solving multi‐objective scaling problems (Qiu et al. 2022). However, its applicability for real‐time detection in embedded systems is limited due to the high requirements on computational resources and storage space (Miao et al. 2025). To solve this problem, this study proposes a lightweight design using ShuffleNetV2 as the backbone network, and the model network structure is shown in Figure 6.

**FIGURE 7:**
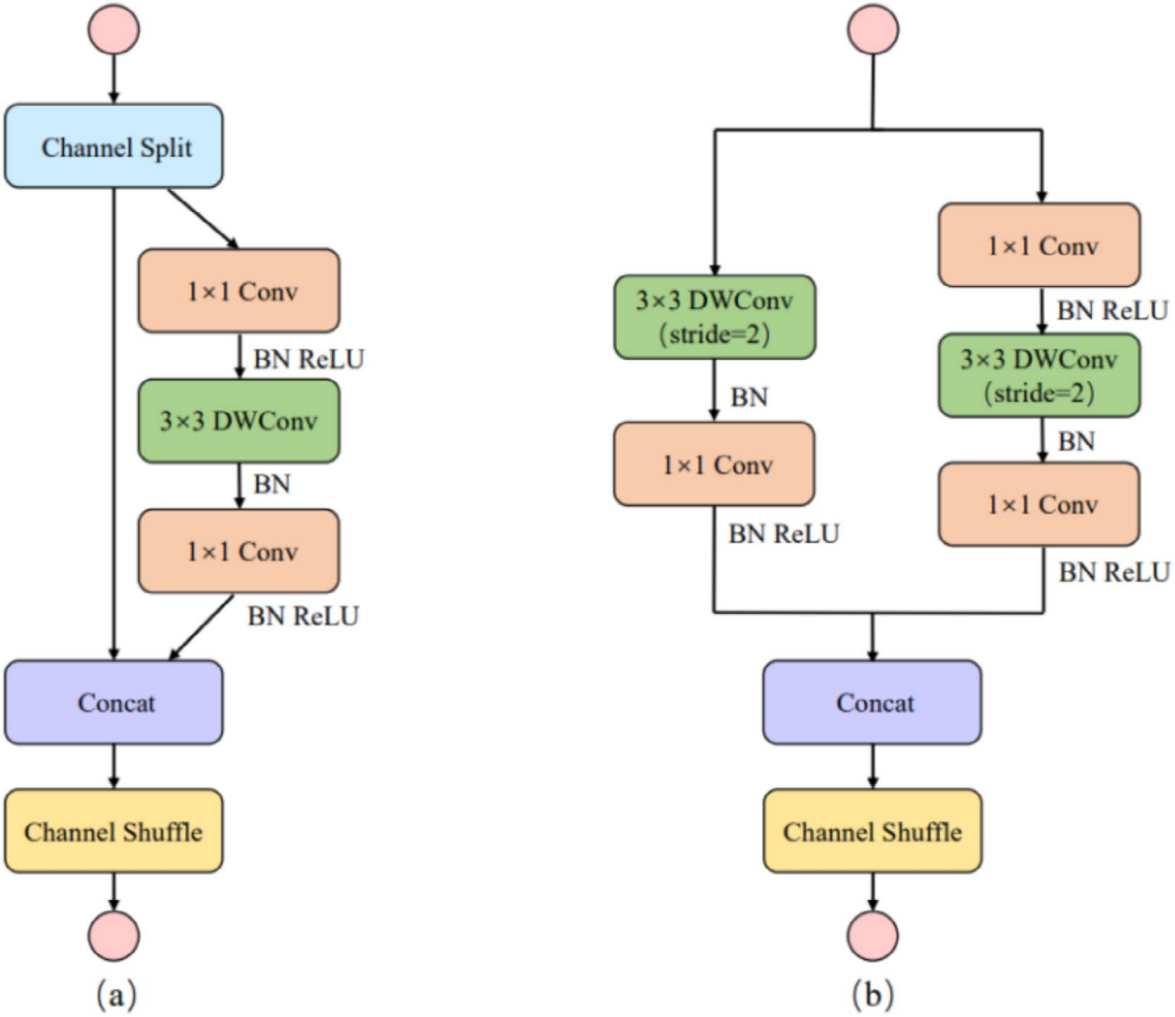
Network structure of ShuffleNetV2. (a) Basic unit; (b) spatial downsampling unit DWConv depth convolution.

To rectify the shortcomings of ShuffleNetV1, ShuffleNetV2 introduced a novel operation named Channel Split. Specifically, at the onset, the input feature map is partitioned into two branches along the channel dimension, with the number of channels in the two branches being C′ and C–C′ respectively. In practical implementation, C′ = C/2. The left‐hand branch conducts an identity mapping, while the right‐hand branch consists of three consecutive convolutions, where the number of input channels is equal to that of output channels. Meanwhile, the two 1 × 1 convolutions are no longer grouped convolutions, and the two branches are effectively divided into two groups. The outputs of the two branches are not added element‐by‐element but concatenated. Subsequently, a Channel Shuffle operation is performed on the concatenated result of the two branches to ensure information exchange between them. Moreover, the concatenation and Channel Shuffle operations can be integrated with the Channel Split of the next module unit into an element‐level operation. Regarding the down‐sampling module, there is no Channel Split. Instead, each branch directly replicates the input, and each branch undergoes down‐sampling with a stride of 2. After final concatenation, the spatial size of the feature map is halved, while the number of channels is doubled. This design not only maintains high efficiency and a lightweight architecture but also enhances the feature‐extraction capability, rendering it suitable for devices with limited resources. The network structure of ShuffleNetV2 is depicted in Figure 7.

**FIGURE 8:**
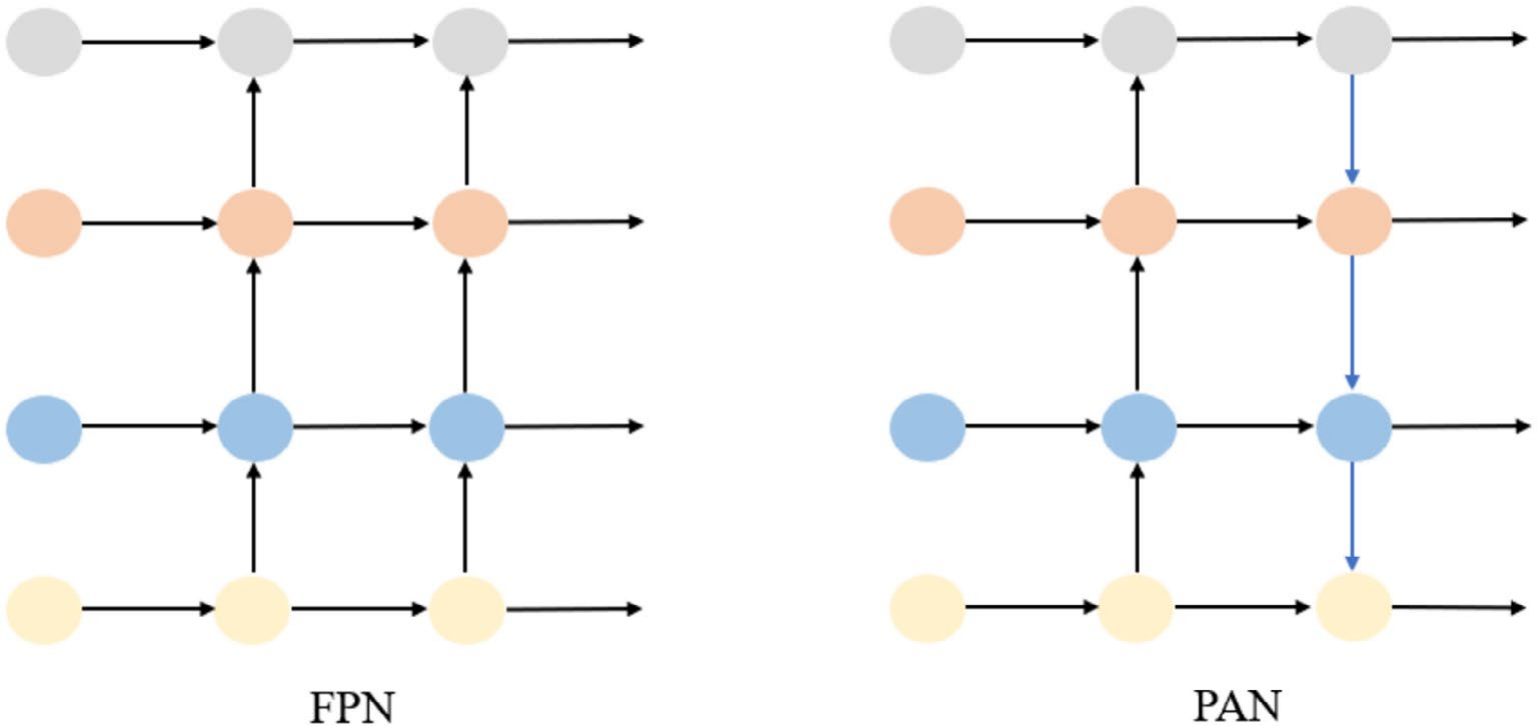
Structures of FPN and PAN.

The Neck module is mainly responsible for further processing the features extracted from Backbone to enhance the multi‐scale information of the features, which in turn provides richer feature inputs to Head. This module consists of two parts: Feature Pyramid Network (FPN) + Path Aggregation Network (PAN). FPN is used to fuse different scales of feature maps from Backbone, and up‐sampling to enlarge the high‐level feature maps step by step, and sum them with the low‐level feature maps element by element, so as to effectively combine semantic and detailed information. PAN adds lateral connections to enhance the feature transfer from low level to high level, avoiding information loss and improving the fusion effect of multi‐scale features, especially the detection accuracy of small and large objects. Figure 8 shows the structure of FPN and PAN.

The Head part is composed of three detectors, which detect feature maps of different scales respectively. The output layer usually generates feature maps of three scales, with each scale corresponding to target objects of different sizes. Each feature map contains three important pieces of information: bounding box coordinates, target class probabilities, and target confidences. The prediction part includes Bounding Box Loss and Non‐maximum inhibition (NMS).

#### 2.3.3 The Training Part of the Model

In this experiment, based on the source code officially provided by YOLOv5, we used the preset configuration file to conduct improved training of the object‐detection model.

To optimize the model parameters, we selected the default optimizer settings of YOLOv5, using the Stochastic Gradient Descent (SGD) optimizer with a default learning rate of 0.01 and fine‐tuned it to improve the training efficiency. In some experiments, we also tried using the Adam Adaptive Moment Estimation (Adam) optimizer.

**FIGURE 9:**
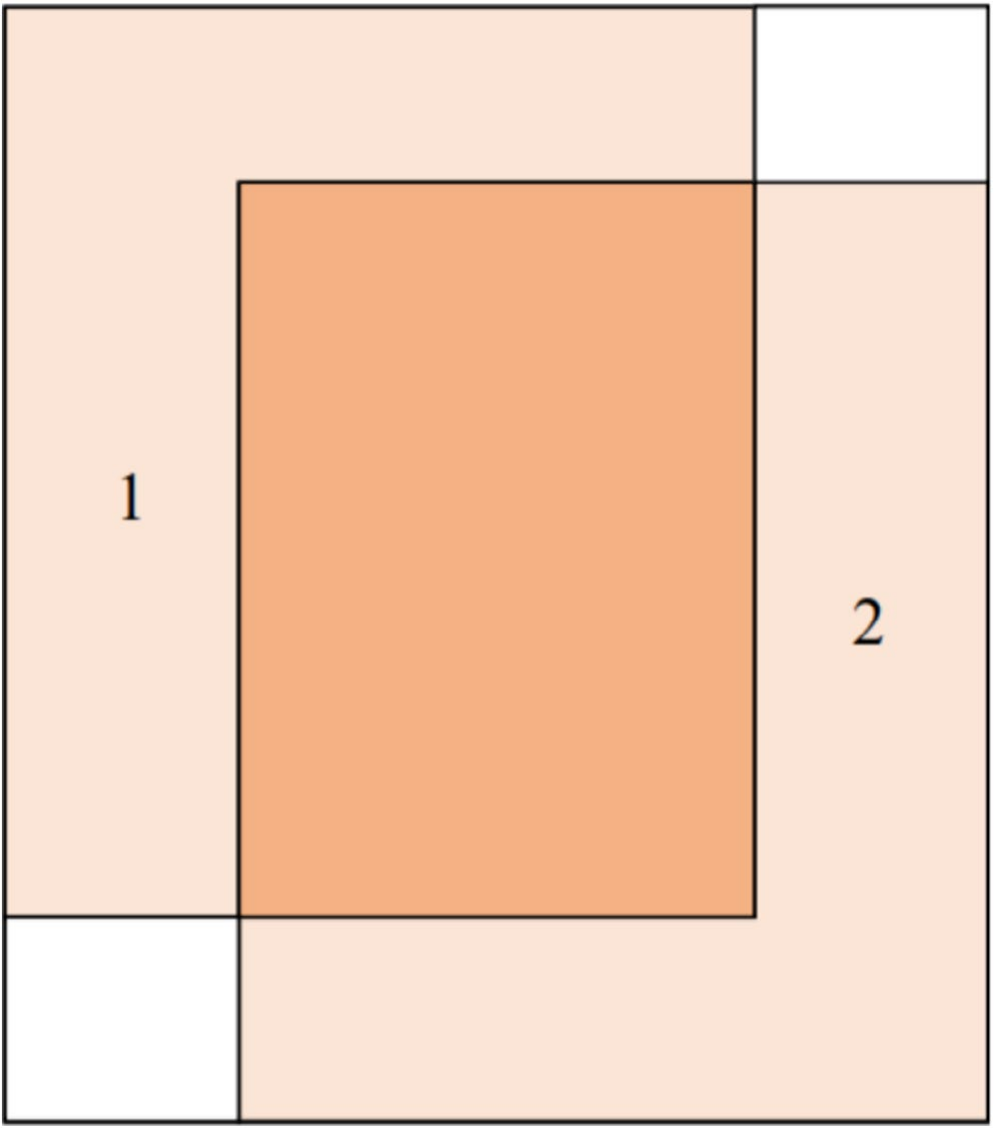
Intersection Over Union.

We used CIOU_Loss as the bounding–box regression loss function. Compared with the traditional IOU_Loss, CIOU_Loss not only takes into account the overlap degree of the boxes but also introduces factors such as the distance between the center points and the aspect ratio. In addition, YOLOv5 also adopts weighted NMS Non‐Maximum Suppression (NMS). By weighting the confidences of different detection boxes, it can more accurately select the optimal target boxes, reduce the appearance of redundant boxes, and improve the detection accuracy and efficiency. The schematic diagram of Intersection Over Union (IOU) is shown in Figure 9.

The specific calculation formula of CIOU_Loss is as follows:

(1) $$\text{CIOU}\_\text{Loss}=1-\text{CIOU}=1-\mathrm{IoU}+\frac{\rho 2\left(b,b_{g_{t}}\right)}{c2}+\mathrm{\alpha v}$$

(2) $$\alpha =\frac{v}{\left(1-\mathrm{IoU}\right)+v}$$

(3) $$v=\frac{4}{\pi 2}\left(\arctan\left(\frac{\omega_{g_{t}}}{h_{g_{t}}}\right)-\arctan\left(\frac{\omega_{g}}{h_{g}}\right)\right)2$$

Among them, IoU is the ratio of the intersection area of box 1 and box 2 to their union area. $\rho \left(b,b_{g_{t}}\right)$ is the Euclidean distance between the center points of box 1 and box 2. c is the diagonal distance of the smallest rectangle that can cover both box 1 and box 2. *α* is a hyperparameter, and ***v*** represents the difference in aspect ratios between box 1 and box 2. $\omega_{g_{t}}$, $h_{g_{t}}$ and $\omega_{g}$, $h_{g}$ are the widths and heights of box 1 and box 2 respectively.

To evaluate the model's generalization ability, we used the k‐fold cross‐validation method. The dataset was divided into k subsets, with k‐1 subsets used for training and the remaining 1 subset used for validation. Through multiple iterations, each subset was used as the validation set once. This method effectively reduces the risk of model overfitting and improves the model's generalization ability. Considering compatibility, the dataset in XML format was converted to TXT format. The YOLOv5 model used in this paper was pre‐trained with the TXT dataset. During the training process, the training duration was set to 200 epochs. Parameters such as the initial learning rate, momentum, and weight decay used the original parameters in YOLOv5. In addition, an early‐stopping strategy and a learning rate scheduler were employed during training to prevent overfitting and optimize the training effect.

The computer hardware configuration used for image processing and model training in this experiment is as follows: CPU: Intel (R) Core (TM) i9—10900K CPU@3.70GHz; GPU: NVIDIA GeForce RTX 3090; Memory (RAM): 64GB. The network model was trained under the Windows 10 Professional 64—bit operating system. All program codes were written in Python 3.7, and OpenCV was used for image processing and display.

### 2.4 Research on the Relationship Between Transmission Speed and Recognition Rate

In the real‐time classification and evaluation system of *Agaricus bisporus*, the relationship between transmission speed and recognition rate is one of the key factors for system performance and efficiency. The transmission speed directly affects the smoothness of the image acquisition, processing, and grading process, while the recognition rate determines whether the system can accurately classify and evaluate the size and quality of mushrooms. Therefore, studying and optimizing the relationship between transmission speed and recognition rate is necessary to improve the overall system performance.

#### 2.4.1 Experimental Design

**FIGURE 10:**
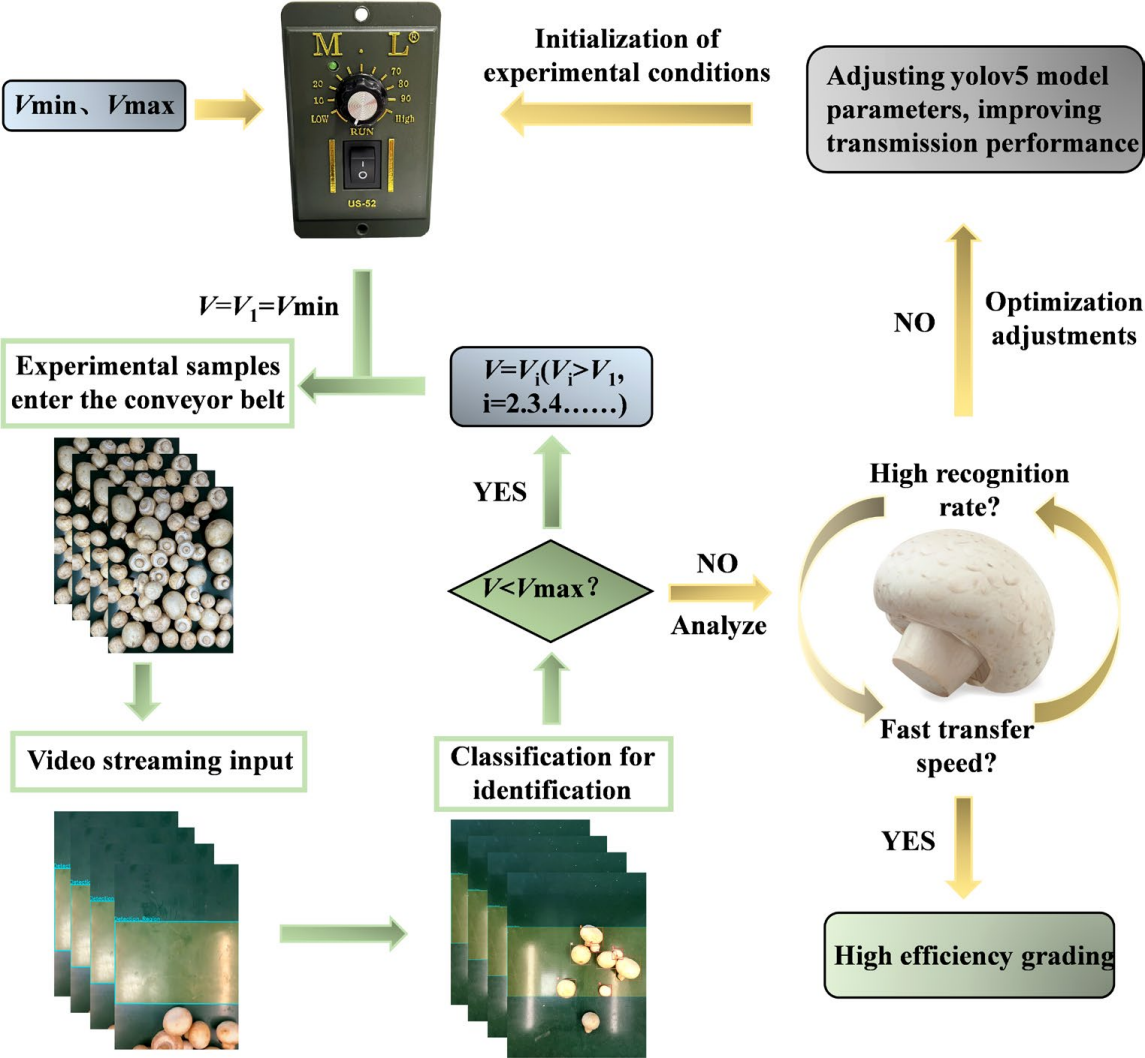
Flowchart of the hypothesis on the relationship between transmission speed and recognition rate.

This experiment aims to investigate the relationship between the transmission speed and the recognition rate of the automatic grading system for *Agaricus bisporus* on the industrial production line. By conducting tests at different conveyor belt speeds, we analyze the impact of speed on the recognition accuracy of the grading system, with the goal of achieving a balance between production efficiency and quality evaluation. Additionally, we analyze whether recognition can be carried out at the maximum transmission speed. To this end, our proposed hypothesis is shown in Figure 10.

**FIGURE 11:**
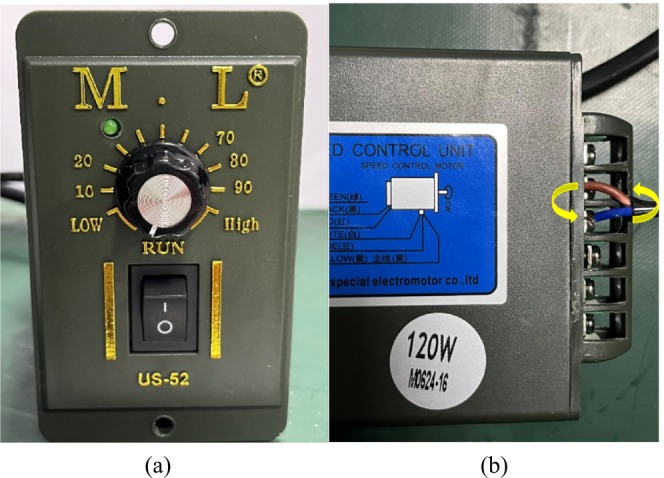
(a) Stepper motor and its gear positions. (b) Schematic diagram of wiring replacement. When the red wire is on the top, the conveyor belt moves forward; when the blue wire is on the top, the conveyor belt moves backward.

In the experiment, while keeping conditions such as illumination, shooting distance, stacking method, and the number of samples constant, the conveyor belt speed was varied. The conveyor belt speed in this experiment was adjusted by successively increasing the gear positions of the stepper motor. There were 11 gear positions in total, and 8 representative and progressively increasing speeds were selected from them, with the speed gradually increasing from 46.83 to 130.17 mm/s. It is worth noting that to ensure that the conveyor belt speed was the sole variable, after completing the shooting of the first set of experimental videos, the wiring of the stepper motor was replaced to reverse the conveyor belt. Meanwhile, the camera was horizontally rotated by 180°. This enabled the shooting of the next set of experimental videos without changing the mushroom stacking method. The specific details of the stepper motor and the wiring replacement are shown in Figure 11. Considering that rotating the camera 180° may cause image distortion, we measured the differences in key features (such as mushroom diameter and position) between the images before and after rotation. The results showed that the image distortion caused by rotating the camera 180° is negligible in practical applications. Finally, 8 sets of experimental videos with different speeds were obtained.

#### 2.4.2 Detection Area Division

**FIGURE 12:**
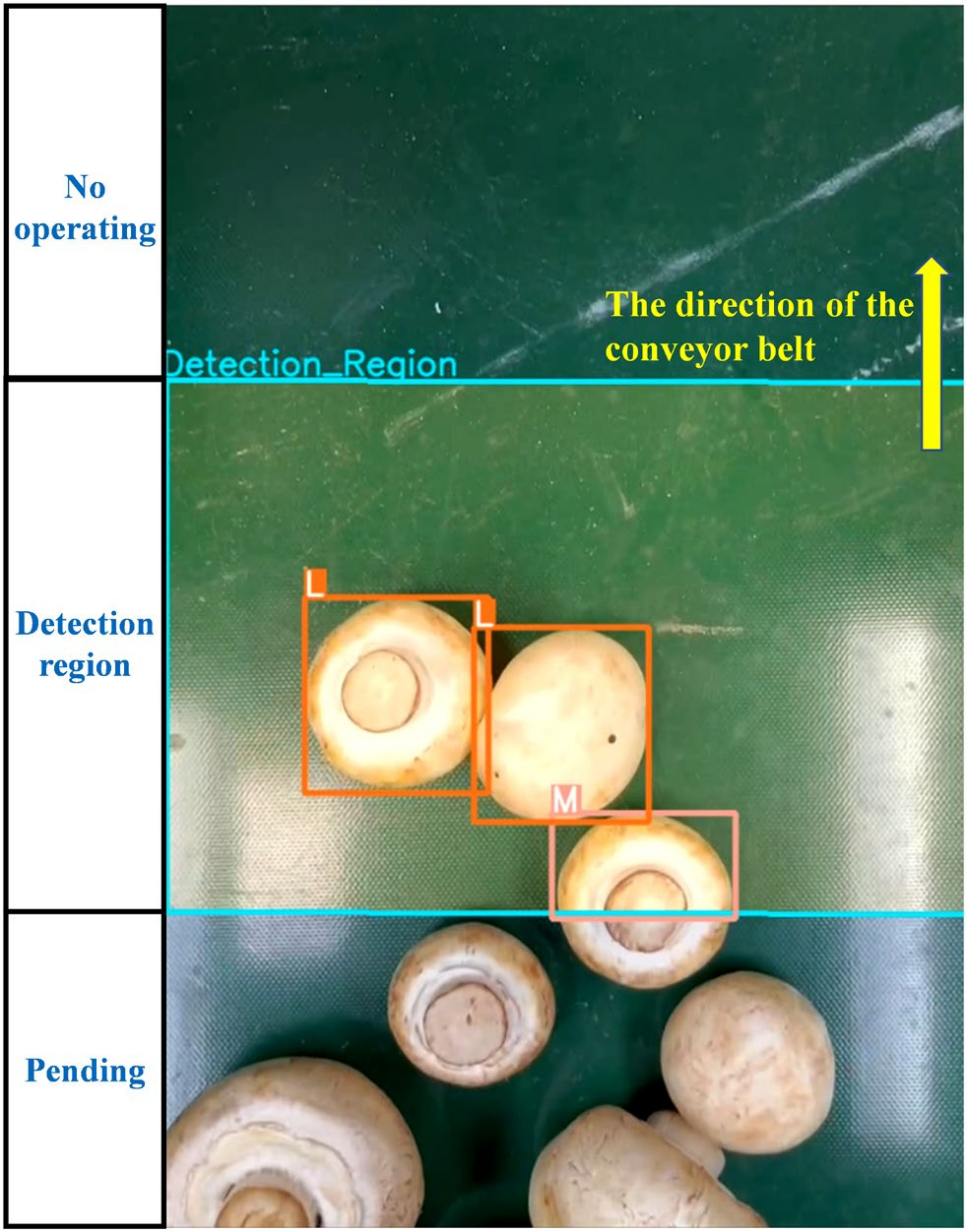
Division of three different sub‐regions.

To simplify the quality–grading procedure, we divided the imaging area into three non‐overlapping sub‐regions, as shown in Figure 12. In the undetermined area, since the *Agaricus bisporus* may not be fully exposed to the camera's field of view, and the lighting conditions are not optimal for high‐quality image recording at this moment, high‐quality image capture is not considered in this area. When the *Agaricus bisporus* enters the detection area, a series of continuous video frames will be taken for it. High‐quality images of each sample will be captured to ensure that each mushroom is evaluated, thereby generating the final quality grade. After the grading is completed, the mushrooms enter the no‐operation area, where no action is taken.

### 2.5 Evaluation Index

To verify the effectiveness of our method, we use Precision, Recall, FPS, and Mean Average Precision (mAP) to evaluate the model of the object detection algorithm. Thecalculations of each indicator are as shown in the equations:

(4) $$\text{Precision}=\frac{\mathrm{TP}}{\mathrm{TP}+\mathrm{FP}}$$

(5) $$\text{Recall}=\frac{\mathrm{TP}}{\mathrm{TP}+\mathrm{FN}}$$

(6) $$\mathrm{AP}=\int_{0}^{1}\text{Precision}\cdot \text{Recall}\;\mathrm{dr}$$

(7) $$\mathrm{mAP}=\frac{\Sigma_{\mathrm{i}=1}^{n}\;AP_{i}}{N}$$

Among them, TP (True Positive) represents the number of positive samples predicted as positive by the model. FN (False Negative) represents the number of positive samples predicted as negative by the model, and FP (False Positive) represents the number of negative samples predicted as positive by the model. *N* is the number of categories, which is equal to 3 in this study.

## 3 Results and Discussion

### 3.1 Results of Model Training

**FIGURE 13:**
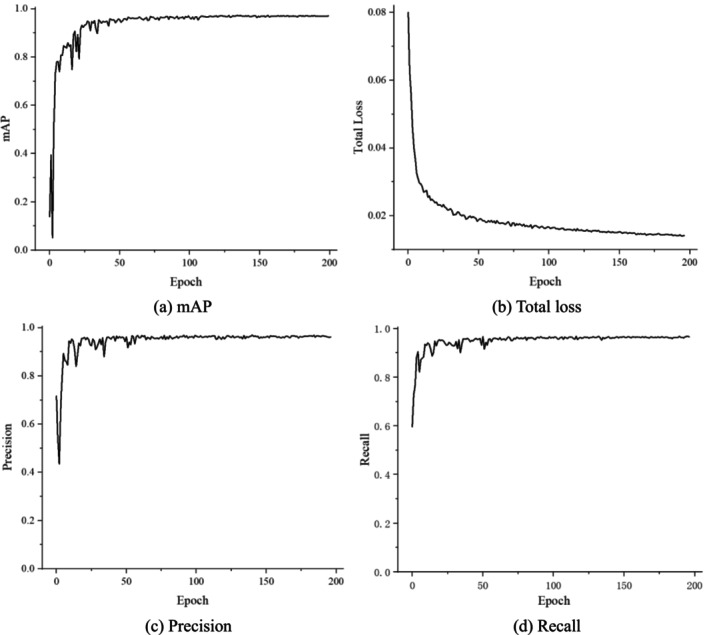
Training result graph.

When training the network, the batch size was set to 16, and the resolution of the images input into the network for training was set to 640 × 640. SGD Stochastic Gradient Descent (SGD) was employed to optimize the parameters of the network model during the training process, and the cosine annealing process was used to adjust the learning rate. The model was trained for 200 epochs on the GPU (Figure 13).

As can be seen from the figure, in the first 50 epochs of training, the loss value decreases rapidly, with mAP, precision, and recall reaching over 90%. When the epochs reach 75, the model begins to converge and the curve gradually flattens. After 175 iterations of the model, the curve has completely flattened, and the results show that mAP reaches 97.1%, precision is 95.4%, recall is 93.7%, and the loss function stays below 0.02, which is within the acceptable range without overfitting. Finally, 200 epochs of training were chosen to end the training.

**FIGURE 14:**
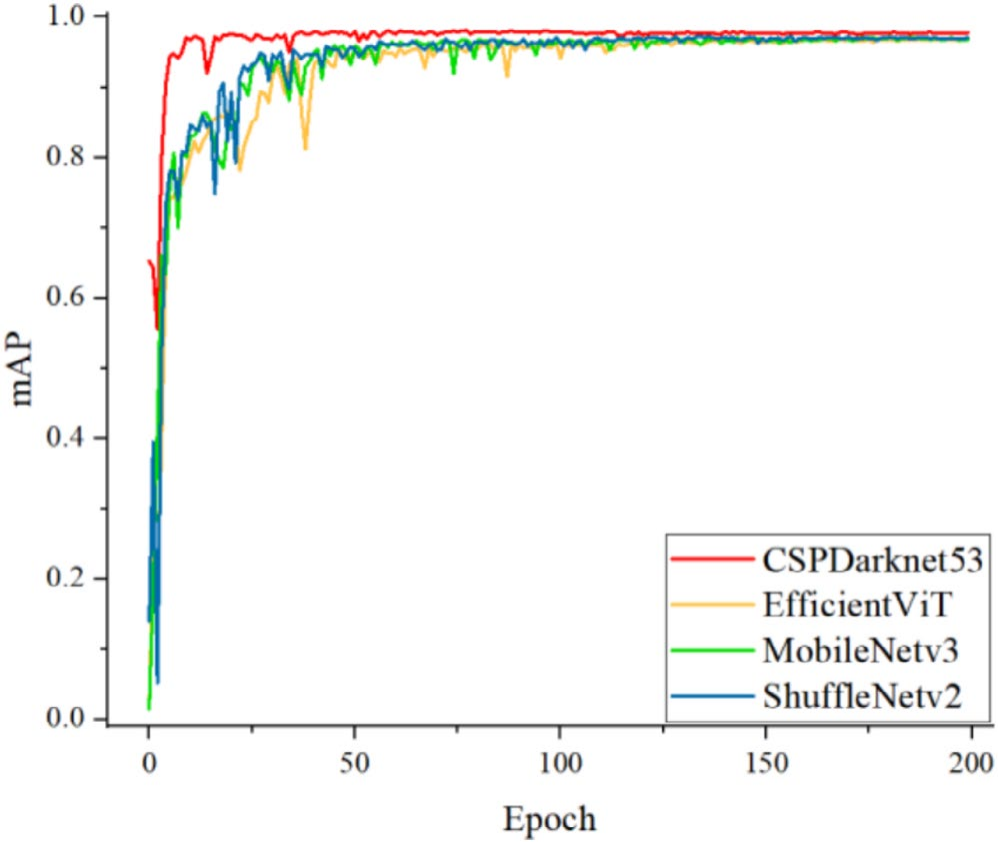
Comparison of map values of Yolov5s with different backbones.

To verify the effectiveness of the lightweight improved model, we introduced two currently popular network architectures, EfficientViT and MobileNetv3, and compared different models. Table 4 presents the experimental results of YOLOv5s trained with different backbones: YOLO‐a employs the EfficientViT model; YOLO‐b utilizes the MobileNetv3 model; YOLO‐c applies the ShuffleNetv2 model. Compared to YOLOv5s, the YOLO‐a model reduces Parameters by 24.3%, Size by 20.1%, and GFLOPS by 32.9%. The YOLO‐b model reduces Parameters by 30%, Size by 28.5%, and GFLOPS by 43.7%, but both models experience a significant decrease in FPS. Compared to YOLOv5s, the YOLO‐c model reduced Parameters by 88.5%, Size by 86.1%, GFLOPS decreased by 88.6%, and FPS increased by 9.8%. Although Precision, Recall, and mAP all decreased compared to the original model, the decline did not exceed 0.7% for any metric, making it negligible. Figure 14 shows the mAP values for YOLOv5s across different backbone models. Experimental results demonstrate that the ShuffleNetv2‐based model reduces parameters and complexity without significantly compromising detection performance, while simultaneously improving FPS detection speed. This achieves a substantial enhancement in video detection capabilities. The significantly reduced lightweight model proves practical for deployment in factory assembly line inspection applications. Considering the balance between model parameters and accuracy, the YOLOv5s lightweight model is both effective and feasible.

**TABLE 4:** Comparison of results of YOLOv5s with different backbones.

Model	Backbone	Precision (%)	Recall (%)	Map_@0.5_ (%)	Parameters (M)	Size (M)	FPS	GFLOPS (G)
YOLOV5s	CSPDarknet53	96.1	94.0	97.7	7.0	14.4	51	15.8
YOLO‐a	EfficientViT	93.1	92.3	96.3	5.3	11.5	41	10.6
YOLO‐b	MobileNetv3	95.3	92.6	95.2	4.9	10.3	35	8.9
YOLO‐c	ShuffleNetv2	95.4	93.7	97.1	0.8	2.0	56	1.8

### 3.2 Dynamic Test

In this paper, a dynamic test of the system was carried out to verify the performance and practicality of the evaluation system for the efficient and real‐time grading of *Agaricus bisporus* in factory settings. The dynamic test mainly includes the evaluation of the system's adaptability in the actual production environment, real‐time testing, and the verification of grading accuracy.

The system was tested against the background of a conveyor belt under three lighting conditions: low light, medium light, and strong light to simulate the common environmental changes in factory production. During the test, *Agaricus bisporus* mushrooms of different specifications and appearances were fed into the visual evaluation system via the conveyor belt, and the image information of the mushrooms was captured in real‐time by a camera. To verify the grading performance of the YOLO model in real‐time videos, we conducted an evaluation through 12 sets of experiments. Each set of experiments was carried out with a random transmission speed, random stacking method, and random quantity. Under the three lighting conditions, 4 sets of experiments were conducted under the same lighting condition. Finally, the actual values of grading were determined using a vernier caliper to evaluate the accuracy of the results. The experimental test results are shown in Table 5.

**TABLE 5:** Dynamic test results of *Agaricus bisporus*.

Intensity of LED illumination (lx)	No.	Grade	System	Caliper	Duration (second)	Accuracy (%)	Miss detection rate (%)	False detection rate (%)
Low light (100)	1	S	0	0	35	98.73	0	1.30
		M	54	53				
		L	25	26				
	2	S	0	0	17	100.00	0	0
		M	45	45				
		L	24	24				
	3	S	0	0	20	98.75	0	1.25
		M	64	63				
		L	16	17				
	4	S	6	9	13	94.05	5.95	0
		M	62	64				
		L	11	11				
Medium light (300)	5	S	7	8	22	98.15	1.85	0
		M	31	31				
		L	15	15				
	6	S	6	6	8	100.00	0	1.89
		M	27	27				
		L	21	20				
	7	S	5	5	9	98.11	1.89	1.89
		M	29	30				
		L	19	18				
	8	S	6	7	7	98.00	2.00	2.00
		M	24	23				
		L	20	20				
High‐intensity light (1000)	9	S	6	6	23	100.00	0	1.32
		M	36	35				
		L	35	35				
	10	S	7	7	10	98.36	1.64	1.64
		M	32	33				
		L	22	21				
	11	S	12	10	6	100.00	0	4.26
		M	22	22				
		L	15	15				
	12	S	5	3	29	98.59	1.41	4.23
		M	45	46				
		L	23	22				

As can be seen from the table, under random transmission speeds, the average automatic grading speed is 230.65 mushrooms per minute. Meanwhile, the average accuracy rate reaches 98.56%, with the average miss detection rate at 1.23% and the average false detection rate at 1.65%. Simultaneously, the information in the table shows that the system developed in this study can conduct detections under all three lighting conditions. In a low light environment, the average accuracy rate can reach 97.88%. The test results demonstrate that the system can accurately identify the appearance features of mushrooms in a complex environment with low resolution and dim light, and accurately classify them according to the established standards.

### 3.3 Relationship Between Transmission Speed and Recognition Rate

**FIGURE 15:**
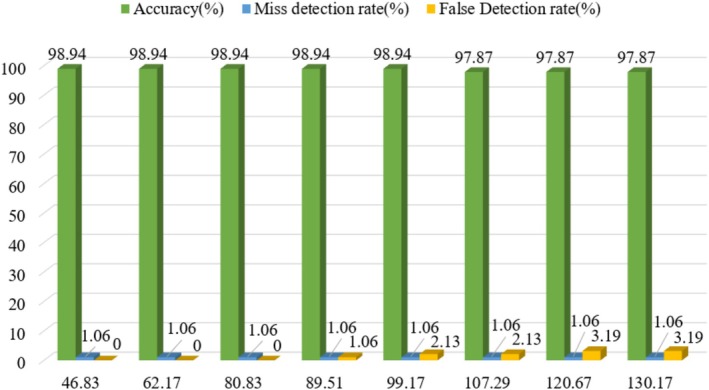
Detection accuracy at different detection speeds (mm/s).

When designing an evaluation system for real‐time automatic grading, the relationship between the transmission speed and the recognition rate is one of the key factors in optimizing the system performance. To analyze this relationship in depth, in this study, 8 groups of real‐time video data with the transmission speed as the sole variable were collected and analyzed. Each group of videos contained 94 mushroom samples. With other conditions (camera resolution, lighting, stacking pattern, etc.) remaining constant, only the transmission speed of the conveyor belt was adjusted. The experimental results are shown in Figure 15.

As can be seen from Figure 15, with the increase in transmission speed, the accuracy rate decreases. The miss detection rate remains unchanged, while the false detection rate gradually increases. The reason is that at higher transmission speeds, more motion blur and image distortion occur. The rapid movement of mushrooms significantly increases the difficulty for the system to capture images. The system's grading ability for mushrooms around the 45 mm threshold (the “M” grade for 44 mm mushrooms and the “L” grade for 46 mm mushrooms) declines. Meanwhile, the motion blur increases the cases where the system mistakenly identifies the mushroom stalks as the “S” grade, thus leading to an increase in the false detection rate. Excessively high conveyor speeds compromise mushroom recognition accuracy. Conversely, while slower conveyor speeds may improve recognition accuracy, they reduce system processing efficiency, creating bottlenecks in the production line. The relationship between the transmission speed and the detection accuracy is not a simple linear one; instead, there exists a transmission speed range within which the detection accuracy can reach a relatively optimal level. Therefore, after analysis, we selected 100 mm/s as the equilibrium point. At this equilibrium point, the system can achieve relatively ideal production efficiency while ensuring a high recognition accuracy rate.

In the 8 sets of experiments, as the transmission speed increased, the system's recognition accuracy remained at 98.94% in the first 5 sets. However, when the transmission speed reached 87.67 mm/s, the recognition accuracy dropped to 97.87% and remained stable in subsequent experiments, indicating that these data represent sustained accuracy rather than peak performance. Although increased transmission speed impacts accuracy, grading efficiency improved. The system maintained grading capability at a maximum transmission speed of 130.17 mm/s, achieving an automatic grading rate of 1066.67 mushrooms per minute with a grading accuracy of 97.87%. This is encouraging and provides further reference value for realizing large‐scale, high‐efficiency mushroom grading in factory settings.

### 3.4 Practicability of the Proposed Approach

The method proposed in this study has considerable accuracy in complex environments while being able to detect mushrooms using a low‐resolution camera. In existing studies (Table 1), the watershed algorithm was used for size grading of *Agaricus bisporus*, achieving an average maximum grading speed of 102.41 units per minute and a grading accuracy rate of 97.42%. However, the speed is relatively slow and far from meeting the requirements of industrial‐scale production (Wang et al. 2018; Jiang et al. 2022). At the same time, there have also been studies on mushroom diameter measurement and positioning, but these methods lack the ability to process multiple targets and do not take into account identification capabilities in complex factory environments (Chen, Yi, et al. 2023; Shi, Mo, et al. 2024). Yolo5, as a single‐stage detector, maintains high accuracy while achieving extremely fast detection speeds and demonstrating strong robustness to changes in lighting and occlusion, making it suitable for real‐time applications. Currently, Yolo algorithms achieve recognition accuracy of 98% in the field of button mushroom research, but these are primarily static detections of large quantities and do not involve grading (Chen, Wang, et al. 2023; Lu and Liaw 2020). In contrast, the method proposed in this paper has advantages such as low equipment costs, the ability to dynamically determine mushroom grades, and the capability to process multiple mushrooms simultaneously, making it more suitable for large‐scale mushroom detection. At maximum transmission speed, it can achieve 1066.67 mushrooms per minute with a grading accuracy rate of 97.87%, thereby promoting the application of vision‐based detection methods in actual production lines. However, since the transmission speed of the transmission equipment of other researchers or companies may be different from that of the equipment in this study, fine‐tuning of the speed may be required to achieve optimal detection results. In addition, this method has some shortcomings. For example, in Table 5, the high miss detection rate in Experiment 4 is due to partial overlap of mushroom samples after they are placed on the conveyor belt, resulting in mushrooms being obscured. The high false detection rates in Experiments 11 and 12 are caused by the system mistakenly identifying water stains and mushroom stems on the conveyor belt as Grade S mushrooms. In future research, we will further optimize the algorithm model, particularly in the mushroom feature extraction component, by considering the introduction of attention mechanisms to better address occlusion and overlap issues. We will also adopt multi‐focus image fusion technology and real‐time video stream processing technology to enhance classification accuracy and real‐time performance (Hu et al. 2024, 2025). Concurrently, we will explore real‐time tracking technology (Jiang et al. 2025) to perform counting operations within designated non‐operational zones, thereby further addressing the requirements of industrial‐scale grading.

## 4 Conclusions

This research presents a machine vision‐based evaluation system for the efficient, real‐time, and automatic size‐grading of *Agaricus bisporus* in an industrial setting. We designed a grading system which encompasses a conveyor belt for transporting *Agaricus bisporus* and an overhead color camera for conducting size and quality assessments. We replaced the backbone network of the YOLOv5 model with EfficientViT, MobileNetv3, and ShuffleNetV2, respectively, and compared them with the original YOLOv5 network in terms of lightweight performance. Ultimately, we selected ShuffleNetV2 as the backbone network, which significantly improved the model's lightweight performance and processing speed, enabling deployment on edge devices. Additionally, by exploring the relationship between the transmission speed and object recognition accuracy, we proposed an equilibrium point that enables the system to maintain a high recognition rate while ensuring efficient production capacity. Research results indicate that for the improved model, the Size decreases by 86.1%, accompanied by an 88.6% reduction in FLOPS and a 9.8% increase in FPS. Moreover, its mAP50 reaches 97.1%. Furthermore, the proposed method was validated through real‐time videos. The results demonstrate that the average detection accuracy of the proposed method is 98.56%. The system can still perform grading even at a high transmission speed of 130.17 mm/s, with an automatic grading rate reaching 1066.67 mushrooms per minute and a grading accuracy of 97.87%. The system also shows strong adaptability and stability under low‐resolution and low‐light conditions, meeting the requirements of real‐time detection. The method proposed in this study provides a suitable technical solution for large‐scale and rapid detection of the size of *Agaricus bisporus* and surface defects of other foods. Future work will enhance detection accuracy and expand to additional *Agaricus bisporus* grade categories.

## Author Contributions

**Qiyang Shui:** conceptualization, data curation, formal analysis, investigation, methodology, software, validation, visualization, writing – original draft, writing – review and editing. **Fajun Miao:** data curation, formal analysis, software. **Senping Liu:** investigation, supervision, writing – review and editing. **Liang Cao:** funding acquisition, methodology, resources. **Huanyu Jiang:** conceptualization, funding acquisition, resources, supervision, writing – review and editing. **Jinzhu Lu:** conceptualization, funding acquisition, methodology, resources, software, supervision, validation, writing – review and editing.

## Funding

This research was funded by the Sichuan Innovation Team Project of the National Modern Agricultural Industry Technology System, project no. SCCXTD‐2025‐12.

## Conflicts of Interest

The authors declare no conflicts of interest.

## Data Availability

Data will be made available on request.
